# Endometrial pattern predicts pregnancy outcome in single‐blastocyst frozen‐embryo transfer: An analysis of 1383 cycles

**DOI:** 10.1002/rmb2.12599

**Published:** 2024-09-09

**Authors:** Kazutaka Kuramoto, Norio Hamada, Keiko Kawamura, Katsuko Egashira, Seiichi Morokuma, Misako Otsuka, Naomi Yoshioka, Takeshi Kuramoto, Kiyoko Kato

**Affiliations:** ^1^ Department of Obstetrics and Gynecology, Graduate School of Medical Sciences Kyushu University Fukuoka Japan; ^2^ Kuramoto Women's Clinic Fukuoka Japan; ^3^ Department of Health Sciences, Graduate School of Medical Sciences Kyushu University Fukuoka Japan

**Keywords:** ART, blastocyst transfer, pregnancy outcomes, single embryo transfer, ultrasound

## Abstract

**Purpose:**

Several studies investigated endometrial patterns, with respect to pregnancy rates following the transfer of embryos but did not distinguish between single‐ and multiple‐blastocyst procedures. We clarified how the endometrial pattern imaged to transfer a frozen embryo is associated with pregnancy outcomes in single‐blastocyst frozen‐embryo transfer (sbFET).

**Methods:**

Patients ≤35 years who underwent sbFET on the hormone replacement protocol. We analyzed endometrial patterns’ associations with pregnancy outcomes in relation to blastocyst grade and pregnancy‐related factors.

**Results:**

Of the 1383 cycles, 483 were Lf, 840 were partial‐Lf, and 60 were non‐Lf. Leaf pattern (Lf): central echogenic line present and continuous. Overall, decreasing distinctness of the central echogenic line was associated with significantly lower rates of clinical pregnancy (Lf: 70.4%; partial‐Lf: 58.1%; non‐Lf: 28.3%) and live birth (56.3%, 45.5%, and 15.0%) and a higher miscarriage rate (20.0%, 21.7%, and 47.1%). Logistic regressions showed pregnancy and live birth to be significantly more likely and miscarriage less likely in Lf than non‐Lf: OR (95% CI): 6.07 (3.24–11.37), 7.43 (3.47–15.39), and 0.20 (0.07–0.57).

**Conclusions:**

Non‐Lf presentation was associated with lower rates of pregnancy and live birth, suggesting it signals unsuitable conditions for embryo transfer. We provide information on the pregnancy outcomes of sbFET for endometrial patterns.

## Abstract 2

Non‐Lf presentation was associated with lower rates of pregnancy and live birth, suggesting it signals unsuitable conditions for embryo transfer of sbFET for all combinations of blastocyst grade and endometrial pattern.

## 1 INTRODUCTION

As a result of estrogen‐dependent angiogenesis and stromal cell proliferation, the endometrium thickens in the late proliferative phase of the menstrual cycle. During this period, on transvaginal ultrasound, the endometrium has a well‐defined “leaf pattern” or “triple‐line pattern,” which consists of a hyperechoic endometrial basal layer, a hypoechoic stromal layer, and a hyperechoic epithelial layer.^1^ The area near the base of the endometrium, where echogenicity is increased, is considered to be a vascular plexus from a histological perspective. Contact between the epithelia of the anterior and posterior endometrial walls increases this echogenicity.^1^ An early study of relationships between endometrial pattern and gynecological disease found that endometrial polyps present with variable echogenicity, disrupting the central line of the leaf pattern, while endometriosis has no effect on endometrial thickness or echo pattern.^2^ Several studies have already investigated endometrial pattern with respect to pregnancy rates following the transfer of fresh or frozen embryos, but do not distinguish between single‐ and multiple‐blastocyst procedures in their analyses; no such work has a scope limited to single‐embryo transfers. It remains unclear whether endometrial pattern can predict clinical pregnancy outcomes; some investigators have suggested positive associations,^3^, ^4^, ^5^ while others have shown no effect.^6^, ^7^, ^8^, ^9^

One study conducted a meta‐analysis on the association between endometrial pattern and pregnancy outcome in women who underwent Intrauterine insemination (IUI). Eight studies reported clinical pregnancy in relation to the endometrial pattern in women undergoing IUI. The triple line pattern assessed on the day of hCG injection was associated with higher clinical pregnancy rates (RR, 1.45; 95% CI: 1.08–1.95; *z* = 2.49; *p* < 0.01; five studies; 1525 cycles). The triple line pattern assessed on the day of IUI was also associated with higher clinical pregnancy rates (RR, 3.21; 95% CI: 1.35–7.61; *z* = 2.64; *p* < 0.008; three studies; 445 cycles).^10^ Studies in assisted reproductive technology (ART) demonstrating associations between endometrial pattern and pregnancy rate often reference a 2010 classification scheme for multiple fresh‐embryo transfer.^7^ It provides for two types of endometrial pattern, as imaged on the day of human chorionic gonadotropin (hCG) administration: (A) broadly hypoechoic, with a well‐defined hyperechoic central line and outer walls (“triple‐line pattern”); (B) isoechoic or homogeneously hyperechoic, with a nonprominent or absent central line.^7^ Endometrial hyperechogenicity has been linked to progesterone elevation, a hallmark of the luteal phase of the menstrual cycle, but it is rarely observed in ART settings because this factor's levels remain low in the proliferative phase of a hormone replacement therapy (HRT) cycle. This discrepancy led us to focus on the endometrium's appearance on ultrasonography on the date of the exam preceding the transfer, which we term the “transfer decision date.”

Since multiple‐embryo transfer is likely to result in multiple pregnancies, which carry risks for both mothers and infants, single‐embryo transfer has come to be preferred by ART clinics. Single‐embryo transfers accounted for 46.0% of cases reviewed in a 2017 meta‐analysis spanning 1382 centers in 39 countries (including both in vitro fertilization; IVF and intracytoplasmic sperm injection; ICSI); in comparison, two embryos were transferred in 49.2% of cases, three in 4.5%, and four or more in 0.3%.^11^

Previous investigations into endometrial patterns and pregnancy rates suffer from several deficiencies. For example, some investigators did not consider endometrial receptivity on the transfer decision date and included cases in which multiple fertilized eggs were transferred. Moreover, the potential confounding effects of embryo quality could not be excluded because the grades of fertilized eggs were not mentioned. Here, we aim to address these gaps in the literature by confining our analysis to FETs of single blastocysts in patients aged ≤35 years. We retrospectively analyzed the association of endometrial pattern with pregnancy outcomes—clinical pregnancy, live birth, and miscarriage—across all cases and separately by embryo grade. We decided to classify endometrial pattern based on its presentation on the transfer decision date and to limit our focus to single‐blastocyst FET, thus providing new knowledge about the usefulness of examining this feature prior to embryo transfer.

## 2 MATERIALS AND METHODS

### 2.1 Subjects

**FIGURE 1:**
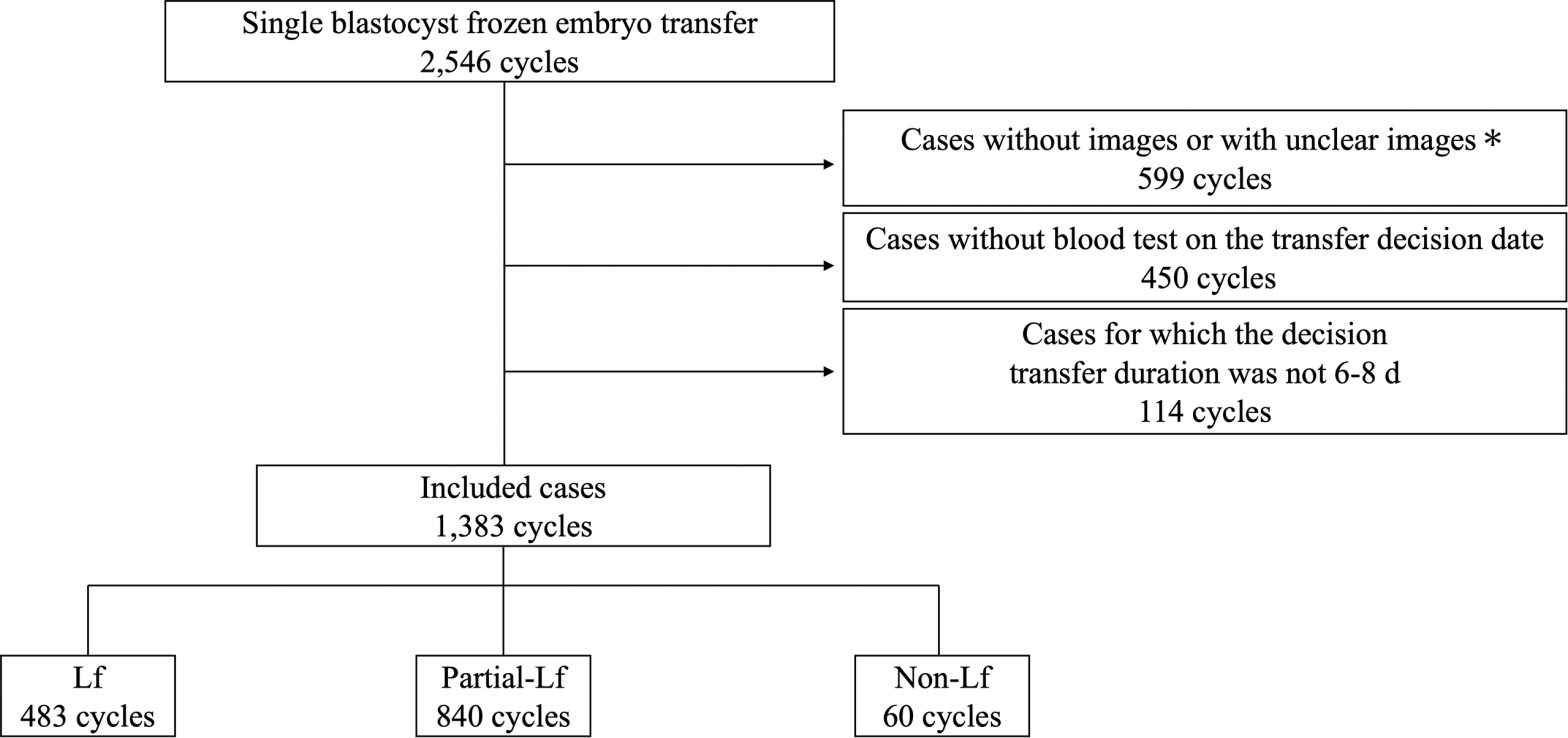
Case selection flowchart. *Unclear images; refer to transvaginal ultrasonographic images captured with the major axis of the uterus parallel to the probe, making it impossible to distinguish the endometrial pattern.

The study population comprised female patients who underwent single‐blastocyst FET at Kuramoto Women's Clinic (Fukuoka, Japan) between February 2012 and December 2019 and were aged 35 years or younger at the time of the procedure. A total of 2546 ART cycles were reviewed for inclusion. Cases were excluded if the endometrium was poorly visualized or if imaging or blood test data were incomplete or missing by transvaginal ultrasound. Cases were further limited to embryo transfers performed 6–8 days after the clinicians' transfer decision date described in further detail below (*n* = 1383 cycles, Figure 1).

We retrospectively analyzed this population for associations between endometrial pattern and pregnancy outcomes—clinical pregnancy, live birth, and miscarriage—including separate sub‐analyses by embryo grade. In each case, blastocysts were cryopreserved at the clinic after oocyte retrieval and fertilization via IVF or ICSI. A single blastocyst was thawed and transferred at an appropriate point in the patient's HRT cycle. All blastocysts transferred were rated 3CC or higher according to the Gardner classification. Patient information was extracted from electronic medical records. No patient received any therapeutic intervention other than the standard procedures described below. Blastocyst quality was graded using the Kuramoto Women's Clinic's criteria, which are based on the Gardner classification. In the Gardner classification, AA corresponds to A, AB, and BA correspond to A', BB corresponds to B, AC, BC, CA and CB correspond to B′, and CC corresponds to C. In AC, BC, CA, and BC of the Gardner classification, if the number of cells is relatively small, B′ is moved down in rank to C in the clinic's classification system. In CC of the Gardner classification system, if the number of cells is very small, the embryo is classified as nontransplantable in the clinic's classification system (Figure S1).

### 2.2 Treatment protocol

Each patient's ovarian stimulation protocol was selected based on their maternal age, cause of infertility, and ovarian response. When at least one follicle exceeded 18 mm in mean diameter, ovulation was induced by administrating either recombinant hCG 5000 IU or hCG 1000–5000 IU + nafarelin acetate hydrate 450–600 μg. Oocytes were retrieved 36 h after ovulation induction and inseminated via either IVF or ICSI (the method was selected in each case based on sperm quality).

Fertilized eggs that developed into blastocysts after 5–6 days of culture were then cryopreserved via vitrification. Embryo transfer was preceded by a transvaginal ultrasound examination to assess endometrial thickness and check for organic disease. If endometrial polyps were suspected based on imaging, a clinician performed a hysteroscopy to confirm this and, if necessary, remove them. If polypectomy was required, embryo transfer was postponed. If focal or diffuse hyperemia, stromal edema, or micropolyps were observed in the uterine cavity, the patient was diagnosed with chronic endometriosis and administered Doxycycline Hydrochloride Hydrate 200 mg/d for 14 days.^12^

The following hormone replacement protocol was applied in all cases: First, the patient was started on estrogen on day 2 or 3 of the menstrual cycle (Estradiol 2.16 mg/every 2 days). On day 12 of the menstrual cycle or later, sagittal uterine images were obtained via transvaginal ultrasound using one of the following systems: SONOVISTA FX (Konica Minolta, Tokyo, Japan), F37 (Hitachi, Saitama, Japan), Voluson E10, or Voluson P8 (General Electric Company, Tokyo, Japan). If endometrial thickness measured ≥7.0 mm in the image, embryo transfer was scheduled for about one week later. If it measured <7.0 mm, the patient was prescribed additional estradiol and scheduled for a second transvaginal ultrasound exam 7–10 days later. The endometrial pattern was classified as described in the next section, based on the image from the first exam in the former case or the second exam in the latter case. Estradiol and progesterone were started 6 days before the date of the planned embryo transfer and continued until the date of the pregnancy test at a minimum. If the course of pregnancy appeared normal, estradiol and progesterone were continued until 8 week 6 days and 9 week 6 days of gestation, respectively.

Embryo transfer success was confirmed via urine immunochromatography at least 16 days after the start of progesterone administration. The presence of a gestational sac in the uterus was classified as a clinical pregnancy. The delivery of a live infant later than 22 weeks of gestation was classified as a live birth. The death of a fetus before 22 weeks of gestation was classified as a miscarriage. In the event of a missed miscarriage, the product of conception was removed via either dilation and curettage (D&C) or manual vacuum aspiration (MVA). The rates of these pregnancy outcomes were calculated as the ratio of the count of each to the total number of embryo transfers in the population of interest.

### 2.3 Endometrial pattern classification

**FIGURE 2:**
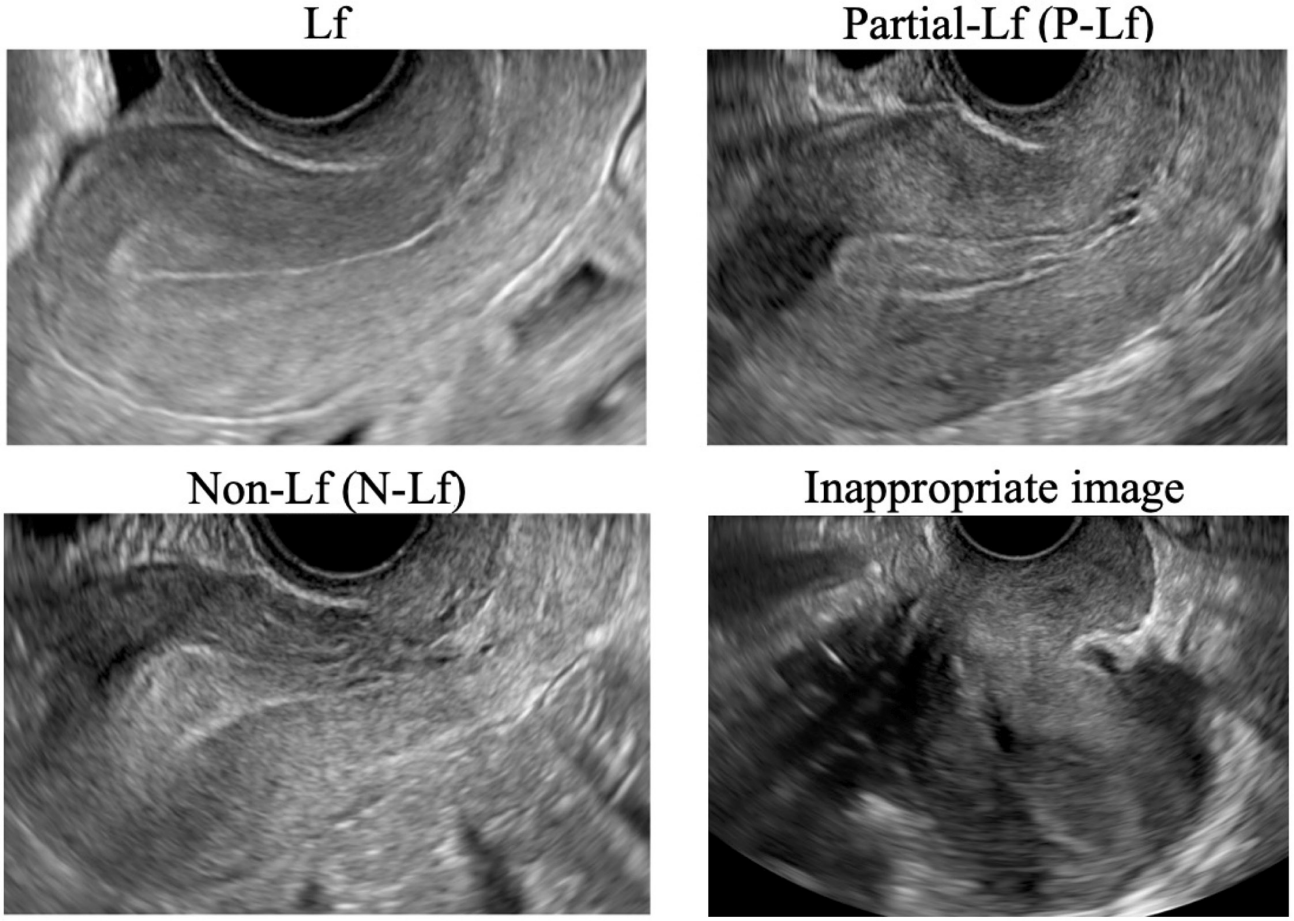
Endometrial pattern classification: Representative ultrasound images. Leaf pattern (Lf): Central echogenic line present and continuous (unbroken) along its full length. Partial‐Lf: Central echogenic line present but discontinuous (broken) in places. Non‐Lf: Central echogenic line of leaf pattern cannot be clearly distinguished. Inappropriate: Endometrial pattern was indistinguishable because the image was captured with the major axis of the uterus parallel to the transvaginal probe.

Endometrial thickness was defined as the maximum distance in the uterine midsagittal plane, as measured in the transvaginal ultrasound image taken on the transfer decision date of the FET cycle. The endometrial pattern was classified as one of three types based on the presence and distinctness of the echogenic line in the center of the classic leaf pattern (Figure 2).

- Leaf pattern (Lf): central echogenic line present and continuous (unbroken) along its full length.
- Partial‐Lf: central echogenic line present but discontinuous (broken) in places.
- Non‐Lf: the central echogenic line cannot be clearly distinguished.

Several technicians were responsible for performing the transvaginal ultrasound examinations, but endometrial patterns were identified from images by the same researcher without knowing the pregnancy outcome. Some images, however, had been taken with the major axis of the uterus parallel to the transvaginal probe, making it impossible to distinguish the endometrial pattern; such cases were labeled “inappropriate” and excluded from the analysis (Figure 2).

### 2.4 Statistical analysis

We used analysis of variance (ANOVA) to compare the averages of patient parameters during the endometrial patterns. Differences in pregnancy, live birth, and miscarriage rates attributable to endometrial pattern were detected using pairwise chi‐square tests, both overall and by blastocyst grade. The effects of endometrial pattern on each pregnancy outcome were evaluated via logistic regression analysis and reported as odds ratios (ORs) and associated 95% confidence intervals (CIs). Factors previously linked to implantation and miscarriage outcomes—age, endometrial thickness, blastocyst grade, endometriosis, hydrosalpinx, antiphospholipid antibody syndrome, and uterine evacuation history^13^—were incorporated into the regression models as confounding variables. The number of days between the transfer decision date and the actual procedure (“days to transplant”) was also included as a likely influential factor. In addition, the same analyses were repeated on a subset of single‐blastocyst FETs limited to cycles in which the patient was undergoing the procedure for the first time at our clinic (“first‐time transfers”). Finally, endometrial patterns obtained in successive cycles from the same patient were compared to ascertain whether improvements in individual patients could affect pregnancy outcomes. All statistical analyses were performed using JMP 15 software (SAS Institute, Cary, NC, USA).

## 3 RESULTS

### 3.1 Patient characteristics

The endometrial pattern was classified as Lf in 483 cycles, P‐Lf in 840 cycles, and non‐Lf in 60 cycles out of 1383 total (984 cases) (Figure 1). It was associated with significant differences in transfer decision date (as day of menstrual cycle) and serum estradiol and progesterone levels (all *p* < 0.01), but not in patient age at oocyte retrieval, days to transplant, endometrial thickness, or blastocyst grade. Non‐Lf was more common than other patterns in patients with a history uterine evacuation. Neither hydrosalpinx nor endometriosis was associated with an endometrial pattern (Table 1).

**TABLE 1:** Patient characteristics: Single‐blastocyst FET, all cycles.

	All cycles (*N* = 1383)	Lf (*N* = 483)	P‐Lf (*N* = 840)	Non‐Lf (*N* = 60)	*p*
Age (y)	32.1 ± 2.4	32.0 ± 2.5	32.1 ± 2.4	32.6 ± 2.5	0.1228
BMI (kg/m^2^)	20.5 ± 2.6	20.4 ± 2.4	20.5 ± 2.5	21.3 ± 3.7	0.0350
AMH (ng/ml)	5.2 ± 3.7	5.1 ± 3.8	5.2 ± 3.7	4.9 ± 3.1	0.7393
Transfer decision date (d)	15.3 ± 1.7	15.2 ± 1.6	15.5 ± 1.8	15.5 ± 1.7	0.0094
Days to transplant (d)	6.9 ± 0.6	6.9 ± 0.7	6.9 ± 0.6	6.9 ± 0.6	0.2602
Endometrial thickness (mm)	10.2 ± 2.0	10.3 ± 1.9	10.1 ± 2.0	10.7 ± 2.2	0.0555
Estradiol (pg/mL)	205.7 ± 80.6	213.6 ± 82.3	200.2 ± 75.0	220.2 ± 129.0	0.0054
Progesterone (ng/mL)	0.3 ± 0.1	0.3 ± 0.1	0.2 ± 0.1	0.3 ± 0.2	0.0024
Embryo grade					
A	96 (6.9%)	30 (6.2%)	61 (7.3%)	5 (8.3%)	0.0872
A'	258 (18.7%)	106 (22.0%)	142 (16.9%)	10 (16.7%)	
B	448 (32.4%)	163 (33.8%)	271 (32.3%)	14 (23.3%)	
B′	437 (31.6%)	146 (30.2%)	266 (31.7%)	25 (41.7%)	
C	144 (10.4%)	38 (7.9%)	100 (11.9%)	6 (10.0%)	
Causes of infertility					
Uterine	596 (43.1%)	191 (39.5%)	370 (44.1%)	35 (58.3%)	0.0144
Endometrial polyp	522 (37.7%)	168 (34.8%)	326 (38.8%)	28 (46.7%)	0.1199
Intrauterine adhesions	63 (4.6%)	15 (3.1%)	44 (5.3%)	4 (6.7%)	0.1458
Chronic endometritis	187 (13.5%)	66 (13.7%)	112 (13.3%)	9 (15.0%)	0.9296
Submucosal fibroid	19 (1.4%)	3 (0.6%)	12 (1.4%)	4 (6.7%)	0.0007
Fallopian tubes	233 (16.9%)	72 (14.9%)	147 (17.5%)	14 (23.3%)	0.1868
Endometriosis	191 (13.8%)	64 (13.3%)	114 (13.6%)	13 (21.7%)	0.1941
Hydrosalpinx	47 (3.4%)	11 (2.3%)	35 (4.2%)	1 (1.7%)	0.1418
Ovulation	413 (29.9%)	140 (29.0%)	251 (29.9%)	22 (36.7%)	0.4715
Polycystic ovary syndrome	311 (22.5%)	101 (20.9%)	193 (23.0%)	17 (28.3%)	0.3715
Hypothyroidism	33 (2.4%)	9 (1.9%)	23 (2.7%)	1 (1.7%)	0.5636
Hyperprolactinemia	87 (6.3%)	34 (7.0%)	49 (5.8%)	4 (6.7%)	0.6799
Decreased ovarian reserve	80 (5.8%)	26 (5.4%)	51 (6.1%)	3 (5.0%)	0.8447
Male	929 (67.2%)	325 (67.3%)	562 (66.9%)	42 (70.0%)	0.8835
Cervical	22 (1.6%)	7 (1.5%)	14 (1.7%)	1 (1.7%)	0.9537
Antiphospholipid antibody syndrome	39 (2.8%)	11 (2.3%)	26 (3.1%)	2 (3.3%)	0.6674
Cause unknown	132 (9.5%)	53 (11.0%)	76 (9.1%)	3 (5.0%)	0.2445
Multiple	774 (56.0%)	258 (53.4%)	472 (56.2%)	44 (73.3%)	0.0133
Uterine evacuation history	291 (21.0%)	85 (17.6%)	186 (22.1%)	20 (33.3%)	0.0086

*Note*: Data presented are mean ± standard deviation or number of cases (percentage). Age: Age on transfer decision date. Transfer decision date: Day of the menstrual cycle on the transfer decision date. Days to transplant: Interval from transfer decision date to actual transplant. Endometrial thickness, Estradiol, and Progesterone are measured on the transfer decision date. Uterine: Diagnosed via hysteroscopy or ultrasound before transplant. Fallopian tubes: Diagnosed via ultrasound. Decreased ovarian reserve: Diagnosed based on an AMH level of <1.2 ng/mL. Cervical: Diagnosed based on positivity for anti‐sperm antibody (not tested in all cases). Uterine evacuation history: The method was dilation and curettage (D&C) or manual vacuum aspiration (MVA).

### 3.2 Endometrial pattern and pregnancy outcomes

**FIGURE 3:**
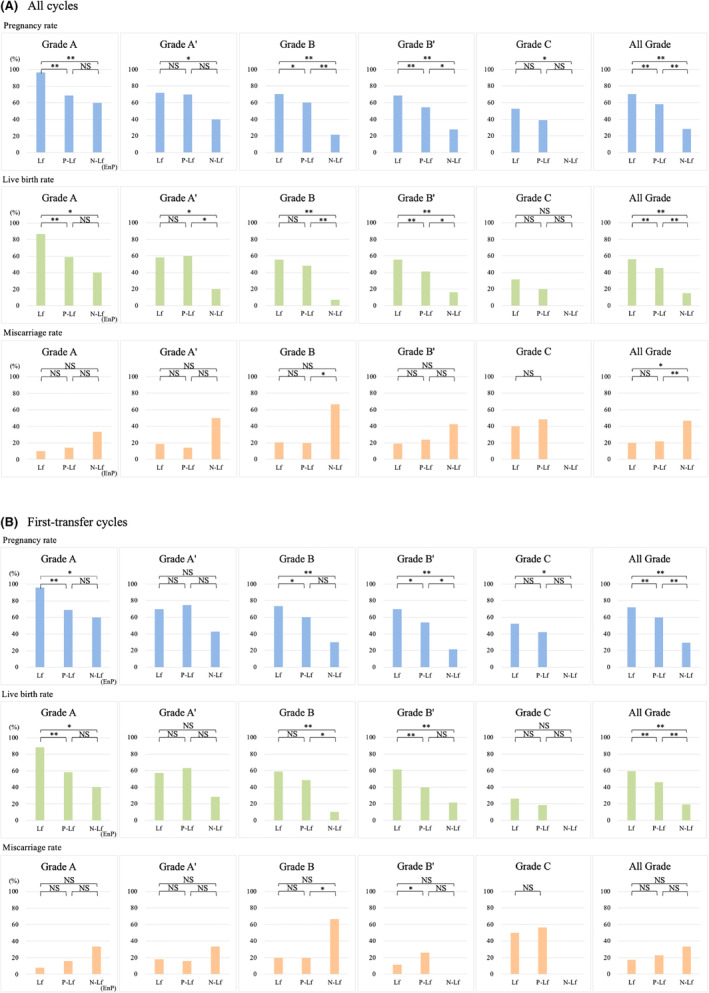
Clinical pregnancy, live birth, and miscarriage rates by endometrial pattern and embryo grade. Tables S1 and S2 are figure guidance. P‐Lf: partial Lf; N‐Lf: non‐Lf; ** *p* < 0.01; * *p* < 0.05; NS: *p* ≥ 0.05. EnP: Endometrial pattern.

Clinical pregnancy rate fell with decreasing distinctness of the echogenic central line (Lf: 70.4%; P‐Lf: 58.1%; non‐Lf: 28.3%; Figure 3A; Table S1). For each blastocyst grade, the pregnancy rate was significantly lower in non‐Lf than Lf cases, and it declined markedly with decreasing embryo quality, from 96.7% in Lf/A cycles (*n* = 30) to 0.0% in non‐Lf/C cycles (*n* = 6). Generally, the pregnancy rate was lower in non‐Lf cases than in P‐Lf cases (*p* < 0.01) and in P‐Lf cases than in Lf cases (*p* < 0.01), but this difference was not significant in every grade. However, it is especially noteworthy that it was significantly higher in Lf cases than in non‐Lf cases in every grade (Figure 3A).

Similarly, the live birth rate fell with decreasing distinctness of the echogenic central line (Lf: 56.3%; P‐Lf: 45.5%; non‐Lf: 15.0%; Figure 3A; Table S1). It was significantly higher in Lf cases than non‐Lf cases overall and in each grade except C (*p* < 0.05) (Figure 3A). As above, it was lower overall in non‐Lf cases than P‐Lf cases (*p* < 0.01) and in P‐Lf cases than Lf cases (*p* < 0.01), but these differences were not significant in every grade.

The findings related to the miscarriage rate were less straightforward. Overall, the miscarriage rate was similar in Lf and P‐Lf cases (20.0% and 21.7%, respectively) but markedly higher in non‐Lf cases (47.1%) (Figure 3A; Table S1). Grade‐specific comparisons tended to show higher miscarriage rates among non‐Lf cases as well, although rarely to a statistically significant degree. Notably, the non‐Lf miscarriage rate exceeded 30% even when morphologically high‐quality embryos (A or A') were used (Figure 3A). We conducted the same analyses on the subset of first‐time transfers at this clinic (*n* = 984 cycles; Figure 3B; Table S2). The endometrial pattern was not associated with significant differences in the grade distribution of embryos transferred (Table S3). Here too, Lf and P‐Lf resulted in higher pregnancy and live birth rates and lower miscarriage rates than non‐Lf, both overall and per grade (although not all differences were statistically significant) (Figure 3B).

### 3.3 Endometrial pattern's associations with pregnancy, live birth, and miscarriage rates by logistic regression analysis

In the multivariate analysis, endometrial pattern was strongly associated with each pregnancy outcome examined (Table 2). Specifically, Lf was associated with a greater likelihood of pregnancy and live birth and a lower likelihood of miscarriage than non‐Lf; the respective ORs (95% CIs) were 6.07 (3.24–11.37), 7.43 (3.47–15.93), and 0.20 (0.07–0.57). Blastocyst grade was also strongly predictive of pregnancy outcomes. Endometrial thickness was associated with clinical pregnancy but not live birth or miscarriage; antiphospholipid antibody syndrome and uterine evacuation history were associated with live birth and miscarriage. Endometrial polyps, adhesions, and chronic endometriosis were associated with pregnancy and live birth. Submucosal fibroid, endometriosis, and hydrosalpinx were not associated with any outcome. For the subset of first‐time transfers, Lf and P‐Lf predicted greater likelihoods of pregnancy and live birth and lower likelihoods of miscarriage than non‐Lf (Table 3). Conversely, antiphospholipid antibody syndrome and uterine evacuation history increased miscarriage risk.

**TABLE 2:** Multivariate analysis: Effects of endometrial pattern on pregnancy outcome in single‐blastocyst FET (all cycles).

Variables	Clinical pregnancy			Live birth			Miscarriage		
	OR	95% CI	*p*	OR	95% CI	*p*	OR	95% CI	*p*
Age (y)	1.00	0.95–1.05	0.9584	1.01	0.96–1.05	0.8004	1.00	0.93–1.07	0.9344
Endometrial thickness (mm)	1.07	1.01–1.14	0.0024	1.05	0.99–1.11	0.1261	1.01	0.92–1.10	0.9054
Transfer decision date (d)	0.94	0.88–1.02	0.1285	1.00	0.92–1.07	0.9070	0.87	0.75–1.00	0.0534
Days to transplant (d)	0.78	0.64–0.94	0.0104	0.92	0.76–1.11	0.4048	0.77	0.57–1.03	0.0807
Estradiol (pg/ml)	1.00	1.00–1.00	0.5787	1.00	1.00–1.00	0.9718	1.00	0.99–1.00	0.5270
Progesterone (ng/mL)	1.26	0.29–5.60	0.7524	1.03	0.24–4.33	0.9694	1.52	0.14–42.19	0.7724
Endometrial pattern									
Lf	6.07	3.24–11.37	<0.0001	7.43	3.47–15.93	<0.0001	0.20	0.07–0.57	0.0024
Partial‐Lf	3.80	2.07–6.99	<0.0001	5.24	2.47–11.10	<0.0001	0.21	0.08–0.59	0.0030
Non‐Lf	Reference			Reference			Reference		
Embryo Grade									
A	5.49	3.00–10.05	<0.0001	8.00	4.38–14.62	<0.0001	0.16	0.07–0.39	<0.0001
A'	3.25	2.09–5.06	<0.0001	4.86	3.01–7.86	<0.0001	0.22	0.11–0.44	<0.0001
B	2.50	1.68–3.72	<0.0001	3.60	2.30–5.62	<0.0001	0.29	0.16–0.54	<0.0001
B′	2.15	1.44–3.20	0.0002	3.08	1.96–4.82	<0.0001	0.32	0.17–0.60	0.0004
C	Reference			Reference			Reference		
Endometrial polyp	0.63	0.49–0.81	0.0003	0.71	0.56–0.92	0.0085	0.96	0.64–1.45	0.8501
Intrauterine adhesions	0.45	0.26–0.78	0.0047	0.32	0.17–0.61	0.0005	2.64	1.08–6.48	0.0341
Chronic endometritis	0.65	0.46–0.93	0.0168	0.62	0.43–0.89	0.0092	1.61	0.91–2.86	0.1010
Submucosal fibroid	0.92	0.35–2.42	0.8609	1.38	0.50–3.77	0.5342	0.54	0.07–4.48	0.5689
Endometriosis	0.90	0.64–1.27	0.5565	0.88	0.63–1.23	0.4498	1.17	0.70–1.94	0.5495
Hydrosalpinx	0.77	0.42–1.42	0.4101	0.94	0.51–1.73	0.8400	0.76	0.24–2.37	0.6352
Antiphospholipid antibody syndrome	1.20	0.60–2.40	0.6060	0.34	0.15–0.77	0.0101	5.59	2.25–13.91	0.0002
Uterine evacuation history	0.86	0.64–1.14	0.2841	0.70	0.52–0.93	0.0129	1.75	1.15–2.67	0.0092

*Note*: We estimated the effects of endometrial pattern on pregnancy outcome (clinical pregnancy, live birth, and miscarriage) after single‐blastocyst FET via logistic regression analysis, incorporating the covariates of embryo grade and other putative predictors. These effects are expressed as unit ORs for patient factors and as relative ORs for endometrial pattern (reference: non‐Lf), embryo grade (reference: grade C), and medical factors (reference: negative status). Patient factors: age, endometrial thickness, transfer decision date (day of menstrual cycle), days to transplant, estradiol, progesterone. Medical factors: endometrial polyp, intrauterine adhesions, chronic endometritis, submucosal fibroid, endometriosis, hydrosalpinx, antiphospholipid antibody syndrome, uterine evacuation history. Abbreviations: CI, confidence interval; OR, odds ratio.

**TABLE 3:** Multivariate analysis: Effects of endometrial pattern on pregnancy outcome in single‐blastocyst FETs (first‐transfer cycles only).

Variable	Clinical pregnancy			Live birth			Miscarriage		
	OR	95% CI	*p*	OR	95% CI	*p*	OR	95% CI	*p*
Age (y)	0.98	0.93–1.04	0.4857	0.99	0.94–1.05	0.7771	1.01	0.92–1.10	0.8820
Endometrial thickness (mm)	1.06	0.99–1.14	0.1082	1.04	0.97–1.11	0.2951	1.01	0.91–1.12	0.8717
Transfer decision date (d)	0.95	0.87–1.04	0.2753	0.98	0.90–1.07	0.6450	0.93	0.79–1.09	0.3711
Days to transplant (d)	0.73	0.58–0.92	0.0083	0.87	0.70–1.09	0.2352	0.82	0.57–1.17	0.2673
Estradiol (pg/ml)	1.00	0.99–1.00	0.3377	1.00	1.00–1.00	0.6784	1.00	0.99–1.01	0.8323
Progesterone (ng/mL)	2.35	0.30–18.52	0.4170	1.32	0.19–9.12	0.7787	1.26	0.02–77.39	0.9135
Endometrial pattern									
Lf	6.43	3.04–13.60	<0.0001	6.16	2.65–14.27	<0.0001	0.31	0.08–1.13	0.0024
Partial‐Lf	4.08	1.97–8.47	0.0002	3.92	1.71–8.97	0.0012	0.40	0.11–1.45	0.0030
Non‐Lf	Reference			Reference			Reference		
Embryo Grade									
A	4.98	2.55–9.72	<0.0001	9.43	4.69–18.98	<0.0001	0.12	0.04–0.31	<0.0001
A'	3.16	1.88–5.32	<0.0001	5.93	3.29–10.68	<0.0001	0.15	0.07–0.34	<0.0001
B	2.38	1.48–3.84	0.0004	4.45	2.54–7.80	<0.0001	0.20	0.09–0.42	<0.0001
B′	1.86	1.15–3.03	0.0121	3.76	2.13–6.65	<0.0001	0.19	0.09–0.41	<0.0001
C	Reference			Reference			Reference		
Endometrial polyp	0.70	0.51–0.95	0.0237	0.86	0.64–1.18	0.3531	0.69	0.41–1.18	0.1763
Intrauterine adhesions	0.44	0.22–0.90	0.0249	0.26	0.11–0.63	0.0028	3.22	0.96–10.85	0.0589
Chronic endometritis	0.67	0.44–1.04	0.0729	0.61	0.39–0.95	0.0287	1.84	0.89–3.78	0.0992
Submucosal fibroid	1.19	0.37–3.85	0.7766	1.36	0.41–4.46	0.6128	0.90	0.10–7.85	0.9239
Endometriosis	0.80	0.54–1.18	0.2531	0.88	0.60–1.30	0.5136	1.03	0.55–1.93	0.9222
Hydrosalpinx	0.74	0.36–1.53	0.4197	0.92	0.45–1.91	0.8245	0.71	0.17–2.92	0.6383
Antiphospholipid antibody syndrome	1.30	0.51–3.32	0.5873	0.12	0.03–0.54	0.0055	18.75	3.89–90.4	0.0003
Uterine evacuation history	1.05	0.72–1.52	0.7983	0.76	0.53–1.10	0.1437	1.85	1.09–3.13	0.0221

Abbreviations: CI, confidence interval; OR, odds ratio.

### 3.4 Within‐patient changes in endometrial pattern in successive cycles

We investigated whether improvements in endometrial pattern in patients graded non‐Lf in one HRT cycle resulted in a live birth (Table 4). Seven cases were non‐Lf at the first transplantation and P‐Lf after the second transplantation, but there were no non‐Lf → Lf cases. We identified six out of seven cases resulting in live births. For these six patients, endometrial thickness was greater in the P‐Lf cycle than the non‐Lf cycle, although not to a statistically significant degree (8.9 ± 3.8 mm vs. 10.2 ± 4.9 mm; results not shown). Embryo grade was identical across cycles in three cases and declined in the other three. Hysteroscopy was performed before the first‐time transfer in three cases, leading to treatment for intrauterine adhesions in one case and endometrial polyp removal in another. Hysteroscopy was performed before the second transfer in the other three cases; the first was treated with endometrial polypectomy, the second was diagnosed with chronic endometriosis and given a 14‐day course of Doxycycline Hydrochloride Hydrate 200 mg/d, and the third was treated with endometrial polypectomy, diagnosed with chronic endometriosis, and given a 14‐day course of Doxycycline Hydrochloride Hydrate 200 mg/d. Thus, endometrial pattern can improve after treatment for organic disease, even among patients initially presenting as non‐Lf.

**TABLE 4:** Clinical details of Non‐Lf → P‐Lf cases.

Case	Age (y)	EnT (mm)	E2 (pg/ml)	Ep	Live birth	Embryo grade	Timing of hysteroscopy
1	30	8.9	145.7	N‐Lf	Not applicable	A'	Before the first transplant Removal of intrauterine adhesions
	30	9.2	135.7	P‐Lf	Applicable	B	
2	34	11.4	177.5	N‐Lf	Not applicable	B′	Before the second transplant Treatment for CE
	34	8.9	148.4	P‐Lf	Applicable	B′	
3	28	8.6	166.2	N‐Lf	Not applicable	B′	Before the first transplant No abnormality
	28	13.4	269.8	P‐Lf	Applicable	C	
4	32	9.9	260.5	N‐Lf	Not applicable	B	Before the second transplant Treatment of CE, endometrial polyp resection
	33	11.1	294.6	P‐Lf	Applicable	B′	
5	30	12	100.9	N‐Lf	Not applicable	B′	Before the second transplant Endometrial polyp resection
	30	17.3	201.9	P‐Lf	Applicable	B′	
6	30	11.4	231.3	N‐Lf	Not applicable	B	Before the first transplant Endometrial polyp resection
	30	11.5	260.9	P‐Lf	Applicable	B	
7	35	8.3	265.0	N‐Lf	Not applicable	B	Before the Second transplant Peel off intrauterine adhesions
	35	7.3	312.8	P‐Lf	Not applicable	B′	

*Note*: Data in the upper and lower rows for each case pertain to the first (non‐Lf) and second (P‐Lf) transfer, respectively. Abbreviations: CE, chronic endometritis; E2, estradiol; EnT, endometrial thickness; Ep, endometrial pattern.

## 4 DISCUSSION

This is the first study to explore how endometrial pattern imaged on the transfer decision date predicts pregnancy outcomes, with a specific focus on single‐blastocyst FET. Previous research has examined such relationships for multiple‐embryo transfers in ART cycles, but their interpretation is complicated by the fact that pregnancy and birth rates correlate with the number of blastocysts transferred. This prompted us to confine our population to single‐embryo transfers and to consider blastocyst grade when analyzing these relationships. Further, since high progesterone reduces endometrial receptivity in the case of fresh‐embryo transfers and affects endometrial pattern, we examined only FETs to eliminate potential confounding effects.^14^

In a recent study of FETs involving at least one blastocyst, Yang et al. analyzed pregnancy outcomes with respect to endometrial pattern evaluated on the first day of progesterone administration. If endometrial thickness was >8 mm at that point, the clinical pregnancy rate was significantly higher in patients exhibiting the classic triple‐line pattern than in those without it, but the corresponding differences in miscarriage and live birth rates were not statistically significant.^15^ However, the fact that the live birth rate was slightly higher among cases with the triple‐line pattern (59.0% vs. 53.8%, *p* = 0.08) suggested to us that the endometrial pattern could be predictive if scrutinized in more detail.

In this study, clinical pregnancy and live birth rates were higher in women with an Lf or P‐Lf pattern on the transfer decision date; also, the miscarriage rate was higher in non‐Lf cases than Lf or P‐Lf cases. In the embryo‐grade‐specific comparisons, the Lf pattern was associated with higher pregnancy and live birth rates and lower miscarriage rates. Isolating the effects of embryo quality barely changed the clear influence of endometrial pattern on pregnancy outcomes. Limiting our scope to single‐blastocyst FETs allowed us to quantify and verify these effects more accurately.

Our findings also clarify the relationships of clinical and medical factors with endometrial pattern. Endometrial pattern was not associated with differences in endometrial thickness. Although there were significant differences in female hormones for each endometrial pattern on the transfer decision date, these differences did not appear to be clinically significant. The reason why logistic analysis also showed that female hormones did not contribute to pregnancy outcome. Therefore, we believe that female hormones cannot be a factor in predicting endometrial pattern or pregnancy outcome.

Across all cycles, non‐Lf was more common than Lf in cases with uterine evacuation history, and the multivariate analysis confirmed that this factor reduced live births and increased miscarriage risk. However, this was not replicated in the first‐time transfer subset, suggesting that the endometrial pattern may have been physically altered by the uterine evacuation procedure itself. Our center used to treat miscarriages with uterine evacuation but has since changed its approach to MVA. The physical trauma caused by intrauterine curettage can cause fibrotic changes in the endometrium, which obstruct the uterine cavity.^16^, ^17^ Non‐Lf presentations may have arisen as the result of endometrial fibrosis and obstruction, which would have affected pregnancy outcomes; however, since we did not investigate miscarriage procedures in detail, this question remains unresolved.

Our study has some limitations. Fearing potential negative effects on endometrial receptivity in patients already suffering from infertility, we did not biopsy endometrial tissue prior to embryo transfer, which meant endometrial patterns could not be analyzed with respect to histopathological features. Also, we did not investigate intrauterine flora. The relationship between endometrial patterns and both histopathological findings and the intrauterine microbiome has not been examined. There are no reports that have investigated these relationships. Therefore, future work should explore how endometrial pattern is related to histopathology and intrauterine flora. One problem with focusing on endometrial ultrasound images from the transfer decision date was variable patient availability, making it impossible to strictly control the decision‐transfer interval. Numerous reports have shown that endometrial thickness affects pregnancy rates in FET cycles,^18^, ^19^, ^20^ but since others refute such a correlation,^9^, ^21^ this question is unresolved. Nevertheless, we attempted to minimize the effects of endometrial thickness by limiting our study population to cases with 6–8 days between decision and transfer, which we determined based on the interquartile range of patient data.

Previous reports on the effects of endometrial pattern on pregnancy outcomes used images taken on the day of progesterone initiation for luteal phase support or on the day of embryo transfer itself. Our decision to focus on the day the decision to transfer was made renders our findings highly practical: clinicians can decide whether to go ahead with embryo transfer based on associations between this endometrial pattern and the grade of the blastocyst to be transferred.

One notable finding is the complete lack of implantations resulting from transfers of Grade C blastocysts in non‐Lf patients. Blastocyst quality is unlikely to be the sole contributing factor since several live births were recorded in non‐Lf women after they improved to P‐Lf following hysteroscopy and appropriate treatment. If a patient rated non‐Lf on the transfer decision date is to receive a low‐grade blastocyst, therefore postponing transfer and considering to perform hysteroscopy and endometritis testing.

## CONFLICT OF INTEREST STATEMENT

The authors declare that there are no conflicts of interest.

## ETHICS STATEMENT

This protocol was approved by the ethics committees of Kyushu University and Kuramoto Women's Clinic (#2020–704).

## HUMAN RIGHTS STATEMENTS

All procedures were carried out in compliance with the ethical standards of the relevant committees on human experimentation, both institutional and national, as well as the 1964 Declaration of Helsinki and its subsequent amendments.

## INFORMED CONSENT

This research is a retrospective study targeting patients who had submitted informed consent for undergoing fertility treatment at our clinic. The opt‐out approach was employed to recruit research participants; this involves providing potential participants with information about the research on our clinic's website and including them as research subjects unless they expressly state their refusal to participate.

## Supporting information


Figure S1.

Table S1.

Table S2.

Table S3.
