# Supplementary material for: Endometrial pattern predicts pregnancy outcome in single‐blastocyst frozen‐embryo transfer: An analysis of 1383 cycles
Source: Reprod Med Biol. 2024 Sep 9;23(1):e12599. doi: 10.1002/rmb2.12599 (PMC11386251; doi:10.1002/rmb2.12599)
Supplement: Supplementary file 1 — Figure S1. [file RMB2-23-e12599-s003.docx]

Figure S1. Blastocyst classification

This research: Classification system used in this study

Gardner: Gardner classification system

In AC, BC, CA, and BC of the Gardner classification, if the number of cells is relatively small, B’ is moved down in rank to C in the clinic’s classification system.

In CC of the Gardner classification system, if the number of cells is very small, the embryo is classified as non-transplantable in the clinic’s classification system.
