# Supplementary material for: Endometrial pattern predicts pregnancy outcome in single‐blastocyst frozen‐embryo transfer: An analysis of 1383 cycles
Source: Reprod Med Biol. 2024 Sep 9;23(1):e12599. doi: 10.1002/rmb2.12599 (PMC11386251; doi:10.1002/rmb2.12599)
Supplement: Supplementary file 2 — Table S1. [file RMB2-23-e12599-s004.docx]

Table S1. Pregnancy outcomes by endometrial pattern and embryo grade in single-blastocyst FET: results data for all cycles

| Grade | EnP | Pregnant | No  pregnancy | All | Pregnancy rate | Lf vs. P-Lf | Lf vs. N-Lf | P-Lf vs. N-Lf |  | Live birth | No  live birth | Live birth rate | Lf vs. P-Lf | Lf vs. N-Lf | P-Lf vs. N-Lf |  | Miscarriage | No miscarriage | Miscarriage rate | | Lf vs. P-Lf | Lf vs. N-Lf | P-Lf vs. N-Lf |
| --- | --- | --- | --- | --- | --- | --- | --- | --- | --- | --- | --- | --- | --- | --- | --- | --- | --- | --- | --- | --- | --- | --- | --- |
| Grade A | Lf | 29 | 1 | 30 | 96.7 | 0.0026 | 0.0067 | 0.6829 |  | 26 | 4 | 86.7 | 0.0078 | 0.0157 | 0.4082 |  | 3 | 26 | 10.3 | 0.6237 | | 0.3792 | 0.2517 |
|  | P-Lf | 42 | 19 | 61 | 68.9 |  |  |  |  | 36 | 25 | 59.0 |  |  |  |  | 6 | 36 | 14.3 |  | |  |  |
|  | N-Lf | 3 | 2 | 5 | 60.0 |  |  |  |  | 2 | 3 | 40.0 |  |  |  |  | 1 | 2 | 33.3 |  | |  |  |
|  | All | 74 | 22 | 96 | 77.1 |  |  |  |  | 64 | 32 | 66.7 |  |  |  |  | 10 | 64 | 13.5 |  | |  |  |
| Grade A' | Lf | 76 | 30 | 106 | 71.7 | 0.7350 | 0.0383 | 0.0520 |  | 62 | 44 | 58.5 | 0.8282 | 0.0193 | 0.0138 |  | 14 | 62 | 18.4 | 0.4440 | | 0.1238 | 0.0523 |
|  | P-Lf | 99 | 43 | 142 | 69.7 |  |  |  |  | 85 | 57 | 59.9 |  |  |  |  | 14 | 85 | 14.1 |  | |  |  |
|  | N-Lf | 4 | 6 | 10 | 40.0 |  |  |  |  | 2 | 8 | 20.0 |  |  |  |  | 2 | 2 | 50.0 |  | |  |  |
|  | All | 179 | 79 | 258 | 69.4 |  |  |  |  | 149 | 109 | 57.8 |  |  |  |  | 30 | 149 | 16.8 |  | |  |  |
| Grade B | Lf | 115 | 48 | 163 | 70.6 | 0.0287 | 0.0002 | 0.0042 |  | 91 | 72 | 55.8 | 0.1307 | 0.0005 | 0.0026 |  | 24 | 91 | 20.9 | 0.8000 | | 0.0588 | 0.0455 |
|  | P-Lf | 163 | 108 | 271 | 60.1 |  |  |  |  | 131 | 140 | 48.3 |  |  |  |  | 32 | 131 | 19.6 |  | |  |  |
|  | N-Lf | 3 | 11 | 14 | 21.4 |  |  |  |  | 1 | 13 | 7.1 |  |  |  |  | 2 | 1 | 66.7 |  | |  |  |
|  | All | 281 | 167 | 448 | 62.7 |  |  |  |  | 223 | 225 | 49.8 |  |  |  |  | 58 | 223 | 20.6 |  | |  |  |
| Grade B' | Lf | 100 | 46 | 146 | 68.5 | 0.0057 | 0.0001 | 0.0112 |  | 81 | 65 | 55.5 | 0.0060 | 0.0003 | 0.0130 |  | 19 | 81 | 19.0 | 0.3403 | | 0.1311 | 0.2639 |
|  | P-Lf | 145 | 121 | 266 | 54.5 |  |  |  |  | 110 | 156 | 41.4 |  |  |  |  | 35 | 110 | 24.1 |  | |  |  |
|  | N-Lf | 7 | 18 | 25 | 28.0 |  |  |  |  | 4 | 21 | 16.0 |  |  |  |  | 3 | 4 | 42.9 |  | |  |  |
|  | All | 252 | 185 | 437 | 57.7 |  |  |  |  | 195 | 242 | 44.6 |  |  |  |  | 57 | 195 | 22.6 |  | |  |  |
| Grade C | Lf | 20 | 18 | 38 | 52.6 | 0.1482 | 0.0161 | 0.0543 |  | 12 | 26 | 31.6 | 0.1500 | 0.1065 | 0.2239 |  | 8 | 12 | 40.0 | 0.5246 | | - | - |
|  | P-Lf | 39 | 61 | 100 | 39.0 |  |  |  |  | 20 | 80 | 20.0 |  |  |  |  | 19 | 20 | 48.7 |  | |  |  |
|  | N-Lf | 0 | 6 | 6 | 0.0 |  |  |  |  | 0 | 6 | 0.0 |  |  |  |  | 0 | 0 |  |  | |  |  |
|  | All | 59 | 85 | 144 | 41.0 |  |  |  |  | 32 | 112 | 22.2 |  |  |  |  | 27 | 32 | 45.8 |  | |  |  |
| All grades | Lf | 340 | 143 | 483 | 70.4 | <0.0001 | <0.0001 | <0.0001 |  | 272 | 211 | 56.3 | 0.0001 | <0.0001 | <0.0001 |  | 68 | 272 | 20.0 | 0.5498 | | 0.0140 | 0.0078 |
|  | P-Lf | 488 | 352 | 840 | 58.1 |  |  |  |  | 382 | 458 | 45.5 |  |  |  |  | 106 | 382 | 21.7 |  | |  |  |
|  | N-Lf | 17 | 43 | 60 | 28.3 |  |  |  |  | 9 | 51 | 15.0 |  |  |  |  | 8 | 9 | 47.1 |  | |  |  |
|  | All | 845 | 538 | 1,383 | 61.1 |  |  |  |  | 663 | 720 | 47.9 |  |  |  |  | 182 | 663 | 21.5 |  | |  |  |

P-Lf: Partial Lf; N-Lf: Non-Lf; Grade: blastocyst grade using the study’s classification system; EnP: endometrial pattern; FET: frozen-embryo transfer

Pairwise chi-square tests were used to identify differences in clinical pregnancy, live birth, and miscarriage rates associated with endometrial pattern, both overall and by embryo grade.
