# Supplementary material for: Endometrial pattern predicts pregnancy outcome in single‐blastocyst frozen‐embryo transfer: An analysis of 1383 cycles
Source: Reprod Med Biol. 2024 Sep 9;23(1):e12599. doi: 10.1002/rmb2.12599 (PMC11386251; doi:10.1002/rmb2.12599)
Supplement: Supplementary file 3 — Table S2. [file RMB2-23-e12599-s002.docx]

Table S2. Pregnancy outcomes by endometrial pattern and embryo grade in single-blastocyst FET: results for first-transfer cycles only

| Grade | EnP | Pregnant | No  pregnancy | All | Pregnancy rate | Lf vs. P-Lf | Lf vs. N-Lf | P-Lf vs. N-Lf |  | Live birth | No  live birth | Live birth rate | Lf vs. P-Lf | Lf vs. N-Lf | P-Lf vs. N-Lf |  | Miscarriage | No miscarriage | Miscarriage rate | Lf vs. P-Lf | Lf vs. N-Lf | P-Lf vs. N-Lf |
| --- | --- | --- | --- | --- | --- | --- | --- | --- | --- | --- | --- | --- | --- | --- | --- | --- | --- | --- | --- | --- | --- | --- |
| Grade A | Lf | 25 | 1 | 26 | 96.2 | 0.0062 | 0.0123 | 0.6757 |  | 23 | 3 | 88.5 | 0.0064 | 0.0120 | 0.4322 |  | 2 | 23 | 8.0 | 0.3636 | 0.1801 | 0.4369 |
|  | P-Lf | 38 | 17 | 55 | 69.1 |  |  |  |  | 32 | 23 | 58.2 |  |  |  |  | 6 | 32 | 15.8 |  |  |  |
|  | N-Lf | 3 | 2 | 5 | 60.0 |  |  |  |  | 2 | 3 | 40.0 |  |  |  |  | 1 | 2 | 33.3 |  |  |  |
|  | All | 66 | 20 | 86 | 76.7 |  |  |  |  | 57 | 29 | 66.3 |  |  |  |  | 9 | 57 | 13.6 |  |  |  |
| Grade A' | Lf | 62 | 27 | 89 | 69.7 | 0.4210 | 0.1442 | 0.0654 |  | 51 | 38 | 57.3 | 0.4076 | 0.1411 | 0.0696 |  | 11 | 51 | 17.7 | 0.7389 | 0.4966 | 0.4154 |
|  | P-Lf | 83 | 28 | 111 | 74.8 |  |  |  |  | 70 | 41 | 63.1 |  |  |  |  | 13 | 70 | 15.7 |  |  |  |
|  | N-Lf | 3 | 4 | 7 | 42.9 |  |  |  |  | 2 | 5 | 28.6 |  |  |  |  | 1 | 2 | 33.3 |  |  |  |
|  | All | 148 | 59 | 207 | 71.5 |  |  |  |  | 123 | 84 | 59.4 |  |  |  |  | 25 | 123 | 16.9 |  |  |  |
| Grade B | Lf | 86 | 31 | 117 | 73.5 | 0.0169 | 0.0039 | 0.0597 |  | 69 | 48 | 59.0 | 0.0723 | 0.0028 | 0.0177 |  | 17 | 69 | 19.8 | 0.9581 | 0.0513 | 0.0464 |
|  | P-Lf | 113 | 75 | 188 | 60.1 |  |  |  |  | 91 | 97 | 48.4 |  |  |  |  | 22 | 91 | 19.5 |  |  |  |
|  | N-Lf | 3 | 7 | 10 | 30.0 |  |  |  |  | 1 | 9 | 10.0 |  |  |  |  | 2 | 1 | 66.7 |  |  |  |
|  | All | 202 | 113 | 315 | 64.1 |  |  |  |  | 161 | 154 | 51.1 |  |  |  |  | 41 | 161 | 20.3 |  |  |  |
| Grade B' | Lf | 69 | 30 | 99 | 69.7 | 0.0102 | 0.0004 | 0.0206 |  | 61 | 38 | 61.6 | 0.0005 | 0.0045 | 0.1784 |  | 6 | 6 | 50.0 | 0.0231 | 0.5316 | 0.3057 |
|  | P-Lf | 88 | 76 | 164 | 53.7 |  |  |  |  | 65 | 99 | 39.6 |  |  |  |  | 17 | 13 | 56.7 |  |  |  |
|  | N-Lf | 3 | 11 | 14 | 21.4 |  |  |  |  | 3 | 11 | 21.4 |  |  |  |  | 0 | 0 |  |  |  |  |
|  | All | 160 | 117 | 277 | 57.8 |  |  |  |  | 129 | 148 | 46.6 |  |  |  |  | 23 | 19 | 54.8 |  |  |  |
| Grade C | Lf | 12 | 11 | 23 | 52.2 | 0.4056 | 0.0326 | 0.0617 |  | 6 | 17 | 26.1 | 0.4196 | 0.1976 | 0.2933 |  | 6 | 6 | 50.0 | 0.6950 | - | - |
|  | P-Lf | 30 | 41 | 71 | 42.3 |  |  |  |  | 13 | 58 | 18.3 |  |  |  |  | 17 | 11 | 60.7 |  |  |  |
|  | N-Lf | 0 | 5 | 5 | 0.0 |  |  |  |  | 0 | 5 | 0.0 |  |  |  |  | 0 | 0 |  |  |  |  |
|  | All | 42 | 57 | 99 | 42.4 |  |  |  |  | 19 | 80 | 19.2 |  |  |  |  | 23 | 17 | 57.5 |  |  |  |
| All grades | Lf | 254 | 100 | 354 | 71.8 | 0.0002 | <.0001 | 0.0001 |  | 210 | 144 | 59.3 | <.0001 | <.0001 | 0.0010 |  | 44 | 210 | 17.3 | 0.0877 | 0.1587 | 0.4059 |
|  | P-Lf | 352 | 237 | 589 | 59.8 |  |  |  |  | 271 | 318 | 46.0 |  |  |  |  | 81 | 271 | 23.0 |  |  |  |
|  | N-Lf | 12 | 29 | 41 | 29.3 |  |  |  |  | 8 | 33 | 19.5 |  |  |  |  | 4 | 8 | 33.3 |  |  |  |
|  | All | 618 | 366 | 984 | 62.8 |  |  |  |  | 489 | 495 | 49.7 |  |  |  |  | 129 | 489 | 20.9 |  |  |  |

P-Lf: Partial Lf; N-Lf: Non-Lf; Grade: blastocyst grade using the study’s classification system; EnP: endometrial pattern; FET: frozen-embryo transfer

Pairwise chi-square tests were used to identify differences in clinical pregnancy, live birth, and miscarriage rates associated with endometrial pattern, both overall and by embryo grade.
