# Supplementary material for: Endometrial pattern predicts pregnancy outcome in single‐blastocyst frozen‐embryo transfer: An analysis of 1383 cycles
Source: Reprod Med Biol. 2024 Sep 9;23(1):e12599. doi: 10.1002/rmb2.12599 (PMC11386251; doi:10.1002/rmb2.12599)
Supplement: Supplementary file 4 — Table S3. [file RMB2-23-e12599-s001.docx]

Table S3. Patient characteristics: single-blastocyst FETs, first-transfer cycles only

|  |  |  | All cycles  (N = 984) | Lf  (N = 354) | P-Lf  (N = 589) | Non-Lf  (N = 41) | p |
| --- | --- | --- | --- | --- | --- | --- | --- |
| Age (y) | | | 32.1±2.5 | 31.9±2.5 | 32.1±2.4 | 32.3±2.6 | 0.1463 |
| BMI (kg/m²) | | | 20.6±2.7 | 20.4±2.5 | 20.6±2.7 | 21.5±4.1 | 0.4585 |
| AMH (ng/ml) | | | 5.1±3.7 | 5.0±3.7 | 5.2±3.7 | 5.0±3.4 | 0.8763 |
| Transfer decision date (d) | | | 15.4±1.7 | 15.2±1.5 | 15.5±1.8 | 15.4±1.1 | 0.1180 |
| Days to transplant (d) | | | 6.9±0.6 | 6.9±0.6 | 6.9±0.6 | 6.8±0.6 | 0.3514 |
| Endometrial thickness (mm) | | | 10.3±2.0 | 10.4±2.0 | 10.2±2.0 | 10.9±2.1 | 0.0078 |
| Estradiol (pg/ml) | | | 203.8±75.6 | 211.9±71.5 | 198.9±78.2 | 204.4±70.8 | 0.0084 |
| Progesterone (ng/ml) | | | 0.3±0.1 | 0.3±0.1 | 0.2±0.1 | 0.3±0.2 | 0.0037 |
| Embryo grade | | |  |  |  |  |  |
|  | A |  | 86 (8.7%) | 26 (7.3%) | 55 (9.3%) | 5 (12.2%) | 0.0703 |
|  | A' |  | 207 (21.0%) | 89 (25.1%) | 111 (18.6%) | 7 (17.1%) |  |
|  | B |  | 315 (32.0%) | 117 (33.1%) | 188 (31.9%) | 10 (24.4%) |  |
|  | B' |  | 277 (28.2%) | 99 (28.0%) | 164 (27.9%) | 14 (34.2%) |  |
|  | C |  | 99 (10.1%) | 23 (6.5%) | 71 (12.1%) | 5 (12.2%) |  |
| Causes of infertility | | |  |  |  |  |  |
|  | Uterine | | 388 (39.4%) | 124 (35.0%) | 244 (41.3%) | 20 (48.8%) | 0.0687 |
|  |  | Endometrial polyp | 336 (34.2%) | 108 (30.5%) | 213 (36.2%) | 15 (36.6%) | 0.1962 |
|  |  | Intrauterine adhesions | 38 (3.9%) | 11 (3.1%) | 23 (3.9%) | 4 (9.8%) | 0.1118 |
|  |  | Chronic endometritis | 124 (12.6%) | 41 (11.6%) | 77 (13.1%) | 6 (14.6%) | 0.7383 |
|  |  | Submucosal fibroid | 13 (1.3%) | 2 (0.6%) | 8 (1.4%) | 3 (7.3%) | 0.0016 |
|  | Fallopian tubes | | 172 (17.5%) | 57 (16.1%) | 107 (18.2%) | 8 (19.5%) | 0.6784 |
|  |  | Endometriosis | 143 (14.5%) | 51 (14.4%) | 85 (14.4%) | 7 (17.1%) | 0.8947 |
|  |  | Hydrosalpinx | 34 (3.5%) | 9 (2.6%) | 24 (4.1%) | 1 (2.4%) | 0.4298 |
|  | Ovulation | | 304 (30.9%) | 107 (30.2%) | 180 (30.6%) | 17 (41.5%) | 0.3246 |
|  |  | Polycystic ovary syndrome | 229 (23.3%) | 77 (21.8%) | 139 (23.3%) | 13 (31.7%) | 0.3452 |
|  |  | Hypothyroidism | 23 (2.3%) | 6 (1.7%) | 16 (2.7%) | 1 (2.4%) | 0.6027 |
|  |  | Hyperprolactinemia | 67 (6.8%) | 26 (7.3%) | 38 (6.5%) | 3 (7.3%) | 0.8627 |
|  | Decreased ovarian reserve | | 62 (6.3%) | 21 (5.9%) | 38 (6.5%) | 3 (7.3%) | 0.9158 |
|  | Male | | 668 (67.9%) | 240 (67.8%) | 400 (67.9%) | 28 (68.3%) | 0.9977 |
|  | Cervical | | 13 (1.3%) | 6 (1.7%) | 7 (1.2%) | 0 (0%) | 0.6042 |
|  | Antiphospholipid antibody syndrome | | 21 (2.1%) | 6 (1.7%) | 14 (2.4%) | 1 (2.4%) | 0.7744 |
|  | Cause unknown | | 101 (10.3%) | 40 (11.3%) | 58 (9.9%) | 3 (7.3%) | 0.6345 |
|  | Multiple | | 542 (55.1%) | 187 (52.8%) | 328 (55.1%) | 27 (65.9%) | 0.2542 |
| Uterine evacuation history | | | 161 (16.8%) | 54 (15.4%) | 100 (17.5%) | 7 (18.0%) | 0.6929 |

Data presented are mean ± standard deviation or number of cases (percentage)
